# Sex Differences in Knee Flexor Strength and Limb Symmetry Across Different Strength Testing Conditions in Healthy Recreational Athletes

**DOI:** 10.3390/jcm15135219

**Published:** 2026-07-03

**Authors:** Natalia Urban, Klara Andrzejczak, Wiktor Witkowski, Maciej Daszkiewicz, Paweł Reichert, Robert Prill, Maciej Kentel, Aleksandra Królikowska

**Affiliations:** 1 Student Research Group of Evidence-Based Healthcare, University Centre of Physiotherapy and Rehabilitation, Faculty of Physiotherapy, Wroclaw Medical University, 50-372 Wroclaw, Poland; natalia.urban@student.umw.edu.pl (N.U.); klara.andrzejczak@student.umw.edu.pl (K.A.); wiktor.witkowski@student.umw.edu.pl (W.W.); 2 Physiotherapy Research Laboratory, University Centre of Physiotherapy and Rehabilitation, Faculty of Physiotherapy, Wroclaw Medical University, 50-372 Wroclaw, Poland; maciej.daszkiewicz@student.umw.edu.pl; 3 Clinical Department and Department of Orthopedics, Traumatology and Hand Surgery, Faculty of Medicine, Wroclaw Medical University, 50-556 Wroclaw, Poland; pawel.reichert@umw.edu.pl; 4 Center of Orthopaedics and Traumatology, University Hospital Brandenburg/Havel, Brandenburg Medical School Theodor Fontane, 14770 Brandenburg, Germany; 5 Faculty of Health Sciences Brandenburg, Brandenburg Medical School Theodor Fontane, 14770 Brandenburg, Germany; 6 eMKa MED Medical Center, 53-110 Wroclaw, Poland; maciej.kentel@emkamed.com.pl

**Keywords:** injury prevention, injury risk reduction, isokinetics, isometrics, muscle strength assessment, limb symmetry index, physiotherapy, rehabilitation, sex differences, sports medicine

## Abstract

**Background:** Normalized strength outcomes and limb symmetry indices (LSIs) are widely used but poorly characterized across testing conditions, and it is unclear if these vary by sex. This study aimed, first, to investigate sex-related differences in normalized knee flexor strength and LSI values across multiple strength-testing conditions in healthy recreational athletes, and, second, to descriptively examine associations among strength outcomes obtained under different testing conditions within female and male participants. **Methods:** In this cross-sectional study, 52 healthy, recreationally active adults (26 females and 26 males) underwent bilateral knee flexor strength testing using three force plate-based isometric assessments, one static dynamometer-based isometric assessment, and three isokinetic dynamometer-based assessments. Differences were analyzed with a mixed analysis of variance (ANOVA), and associations were assessed using Pearson correlations. **Results:** Males showed higher normalized knee flexor strength than females across all testing conditions (main effect of sex: *p* < 0.001; partial η^2^ = 0.334–0.371), with the magnitude of these sex-related differences varying across testing conditions (sex-by-testing condition interaction: *p* < 0.001; partial η^2^ = 0.215–0.230). LSI values did not differ by sex (*p* = 0.896) and remained consistent across testing conditions (*p* = 0.385). Correlations were generally stronger within force plate-based and isokinetic dynamometer-based assessments (r = 0.528–0.922) than between different testing conditions. **Conclusions:** Sex-related differences were observed for normalized knee flexor strength but not for LSI values. Strength outcomes obtained under different testing conditions should not be considered directly interchangeable.

## 1. Introduction

The knee flexors, primarily the hamstrings, play a key role in maintaining knee joint stability, movement control, physical performance, and injury risk. Recent evidence further indicates that neuromuscular control of the lower limb contributes substantially to movement strategies associated with injury risk and remains an important determinant of functional recovery following anterior cruciate ligament (ACL) injury and reconstruction. Therefore, knee flexor strength and limb symmetry are fundamental components of the functional musculoskeletal assessment [1,2,3,4,5,6].

Limb symmetry for a given variable, such as muscle torque or hop test distance, is typically quantified using the Limb Symmetry Index (LSI). This index reflects differences between limbs and is commonly used in clinical and sports settings. Values near 100% indicate higher symmetry between limbs [7,8], while a threshold of ≥90% is commonly used to evaluate readiness to return to sport (RTS) after musculoskeletal injuries [9].

Although LSI is a practical and intuitive measure, its interpretation can be affected by factors such as the variable used for calculation, testing conditions, and other methodological considerations. Furthermore, it does not always accurately reflect the true functional capacity of the limb. Achieving threshold values does not guarantee a safe RTS after an injury or a lower risk of primary or secondary injury. Therefore, it should not be used in isolation, but rather interpreted alongside the underlying data on which it is based, such as strength outcomes. For example, in patients with musculoskeletal disorders, interpreting the LSI without considering absolute strength values, particularly in the presence of contralateral weakness, may lead to an overestimation of recovery [10,11,12,13,14,15,16,17]. Furthermore, normative data on LSI in healthy populations are limited, making it hard to interpret individual results and emphasizing the need for baseline data. Studies in healthy populations are essential for understanding limb symmetry across various tests and establishing reference values for clinical populations.

Knee flexor strength can be assessed using different testing approaches, most commonly isometric and isokinetic protocols. Depending on the testing setup, these assessments may be performed using force plates, handheld dynamometers, fixed-frame (static) dynamometers, or isokinetic dynamometers. The gold standard for muscle strength assessment, offering high reliability and validity in various clinical populations, remains isokinetic dynamometry [18,19,20,21]. However, the high cost and limited accessibility of isokinetic dynamometers restrict their use in many settings. Therefore, alternative approaches, such as isometric assessments performed using force plates or handheld and fixed-frame dynamometers, are becoming increasingly common, although their comparability with isokinetic dynamometry remains unclear and requires further investigation [20,21,22,23,24,25]. These approaches are increasingly used to assess knee flexor strength [26,27]. Consequently, due to differences in contraction type, testing setup, equipment availability, and clinical feasibility, various assessment methods are used across both research and clinical settings, raising questions about the extent to which their results can be meaningfully compared.

Despite well-documented sex-related differences in muscle morphology, strength, and neuromuscular characteristics, it remains unclear whether these differences are reflected in LSI values or in the relationships between different strength assessment methods [28,29,30].

Sex is an important biological variable in muscle strength assessment, as differences in muscle mass and torque-generating capacity may influence absolute strength outcomes, whereas limb symmetry measures appear to be less sex-dependent [31,32,33,34]. However, previous studies have not conclusively determined whether strength and symmetry outcomes are similar across testing modalities in both female and male participants. Furthermore, the use of different assessment methods with varying mechanical and methodological characteristics complicates the interpretation and comparability of strength outcomes across studies and clinical settings.

To address the above-mentioned gaps, the primary aim of this study was to investigate sex-related differences in normalized knee flexor muscle strength and LSI values across multiple strength-testing conditions in healthy recreational athletes. The secondary aim was to descriptively examine associations among strength outcomes obtained under different testing conditions within female and male participants.

It was hypothesized that normalized absolute strength values would differ between sexes across all testing conditions, whereas LSI values would demonstrate smaller or non-significant between-sex differences. Given the limited available evidence, potential sex-related differences in the patterns of associations among strength outcomes obtained across different testing modalities were explored descriptively.

## 2. Materials and Methods

### 2.1. Ethics, Study Design, and Setting

The study was conducted in accordance with the Declaration of Helsinki and approved by the Bioethics Committee of Wroclaw Medical University, Wroclaw, Poland (approval no. KB 351/2025; 9 September 2025). Before participating, all volunteers received detailed information about the study procedures and provided written informed consent.

The study was reported in accordance with the Strengthening the Reporting of Observational Studies in Epidemiology (STROBE) Statement for cross-sectional studies [35,36]. Because the study had an observational cross-sectional design, preregistration was not considered mandatory at the time of study planning. However, it has to be highlighted that all study objectives, eligibility criteria, outcomes, testing procedures, and statistical analyses were defined prior to participant recruitment and data collection [37].

Testing began just after obtaining the aforementioned approval, and the last participant was tested at the end of November 2025. All measurements were taken in a single testing session for each participant, so there was no exposure period or follow-up assessment.

The study was performed in the Physiotherapy Research Laboratory, University Centre of Physiotherapy and Rehabilitation, Faculty of Physiotherapy, and the Clinical Department of Orthopedics, Traumatology, and Hand Surgery; Department of Orthopedics, Traumatology, and Hand Surgery; Faculty of Medicine; Wroclaw Medical University, Wroclaw.

### 2.2. Participants

The volunteers were primarily recruited from students at Wroclaw Medical University. Because volunteer participation from this group was insufficient, additional volunteers were recruited from outside the university, using the same eligibility criteria. Before muscle strength measurements, all participants underwent a medical history interview and physical examination.

Inclusion criteria were: age 20–30 years; no history of injuries or disorders involving the lower limbs or spine; no current pain in the lower limbs or spine; good general physical and mental health; no diagnosed systemic disease; Physical Activity Level (PAL) 1.6–1.8 (moderate to high recreational activity); recreational physical activity only (no competitive/professional sport participation); body mass index (BMI) 18.5–29.99 kg/m^2^; full and symmetrical knee joint range of motion bilaterally; side-to-side differences in knee circumference ≤ 2 cm and thigh circumference < 3 cm, and bilateral muscle strength graded as 5 on the Lovett scale for all muscles acting on the knee joint. The circumference thresholds were selected as pragmatic screening criteria to exclude individuals with potentially clinically relevant asymmetries due to joint effusion, residual swelling, muscle atrophy, or prior lower-limb pathology that could affect strength and limb symmetry measurements. Similar circumference-based screening criteria have been used in previous studies conducted by the given research group [38,39].

Exclusion criteria were: age < 20 or >30 years; any previous lower-limb or spinal injury or disorder; current pain in the lower limbs or spine; poor physical or mental health status; diagnosed systemic disease; PAL < 1.6 or >1.8; competitive/professional sport participation; BMI < 18.5 or >29.99 kg/m^2^; limited knee range of motion; limb circumference asymmetry exceeding the inclusion thresholds; or muscle strength < 5 on the Lovett scale in either limb.

It is worth noting that PAL was assessed using a structured interview during which participants reported the frequency and duration of their regular weekly physical activity. Based on these data, PAL was classified according to predefined criteria. Participants were eligible if their PAL ranged from 1.6 to 1.8, corresponding to moderate-to-high recreational physical activity performed approximately 280–420 min per week.

The dominant limb was defined as the limb that the participant indicated would be used to kick a ball [40].

Participants fulfilling all eligibility criteria were recruited into sex-specific study groups (female and male). For the present analysis, only participants with complete data who successfully completed all strength tests under all planned testing conditions were included (complete-case analysis).

### 2.3. Variables

The primary outcome variables were normalized knee flexor muscle strength values obtained under each testing condition. Specifically, maximal force values were extracted from force plate isometric testing, maximal isometric torque values from fixed-frame static dynamometry, and peak torque values from isokinetic dynamometry. For each condition, strength outcomes were recorded separately for the dominant and nondominant limbs and normalized to body mass, expressed in kgf/kg for force plate testing and Nm/kg for dynamometry-based assessments. All strength variables were treated as continuous outcomes.

Secondary outcome variables included LSI values calculated for each testing condition and the associations between normalized strength outcomes obtained across different testing conditions. LSI was calculated as the ratio of nondominant- to dominant-limb strength × 100, with the dominant limb as the reference; values closer to 100 indicate greater inter-limb symmetry. Associations between normalized strength outcomes were examined separately within female and male participants.

The primary independent variable was sex (female vs. male), treated as a categorical between-subject factor. Testing condition (seven strength-testing conditions) was treated as a within-subjects categorical factor in the statistical analyses. Strength outcomes were analyzed separately for the dominant and nondominant limbs. Additional descriptive variables included age, body mass, body height, and BMI. These variables were summarized descriptively to characterize the study sample. No covariate adjustment was applied in the primary analyses, as the study aimed to examine sex-related differences under standardized testing conditions in a relatively homogeneous population of healthy recreational athletes, and all strength outcomes were normalized to body mass. All variables were defined a priori and measured using standardized testing procedures.

### 2.4. Measurements

All measurements were performed during a single testing session that included three groups of tests, namely force plate-based, static dynamometer-based, and isokinetic dynamometer-based tests of knee flexor muscle strength under seven different testing conditions.

Each testing condition was applied bilaterally. The order of testing conditions and the starting limb were determined using a pre-generated randomization schedule for all 52 participants. All possible sequences of the three groups of testing conditions were included and distributed as evenly as possible across the study sample. For each sequence, predefined allocation slots were created, with approximately balanced representation of assessments starting from the dominant and nondominant limbs. Participants were assigned consecutively according to the pre-generated randomization list. This procedure was used to minimize potential order, fatigue, and learning effects associated with repeated measurements.

To reduce the potential effect of fatigue, rest periods of approximately 30 min were scheduled between successive test groups. These intervals were extended as needed when a participant reported excessive fatigue.

Prior to data collection, participants completed a standardized warm-up and were familiarized with each testing procedure. Practice trials were conducted for all testing conditions prior to the recorded measurements. During testing, consistent verbal instructions and encouragement were provided using standardized commands.

Measurements were conducted by trained examiners according to a predefined protocol detailed in a related study [38] and under the supervision of an experienced researcher to ensure procedural consistency. After completion of all testing procedures, participants performed standardized stretching exercises targeting the tested muscle groups. A summary of the testing setup and conditions is provided in Table 1. During all the tests, the ankle of the tested limb was in a neutral position.

**Figure 1:**
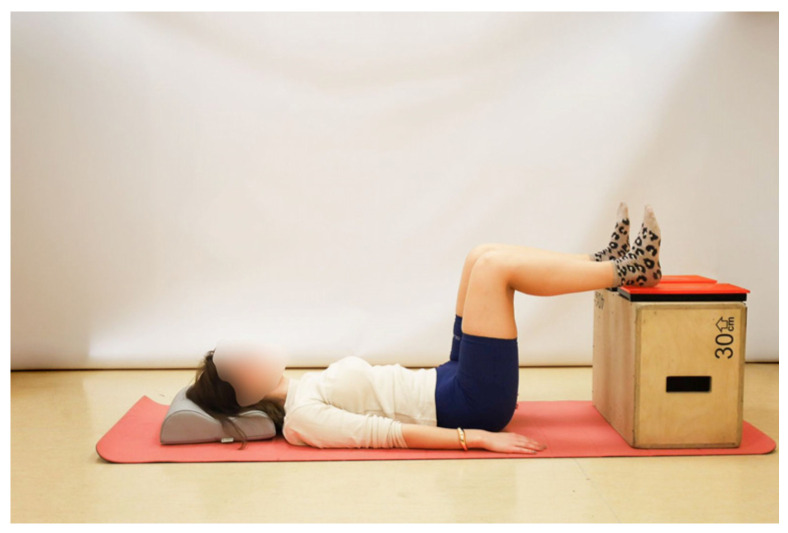
Force plate isometric test (FP-IM-90/90) setup: supine position; hip and knee flexed to 90°; heel placed on an elevated support with the force plate.

**Figure 2:**
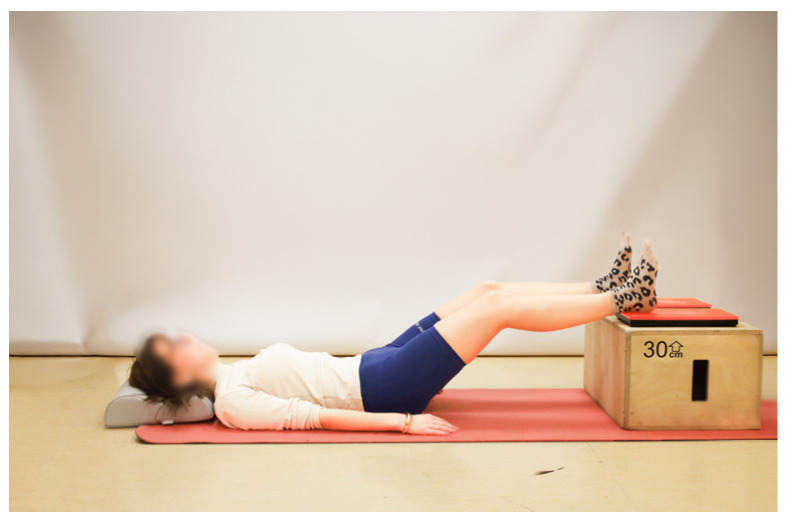
Force plate isometric test (FP-IM-30-30 cm) setup: supine position; knee flexed to 30°; heel placed on a 30 cm elevated support with the force plate.

**Figure 3:**
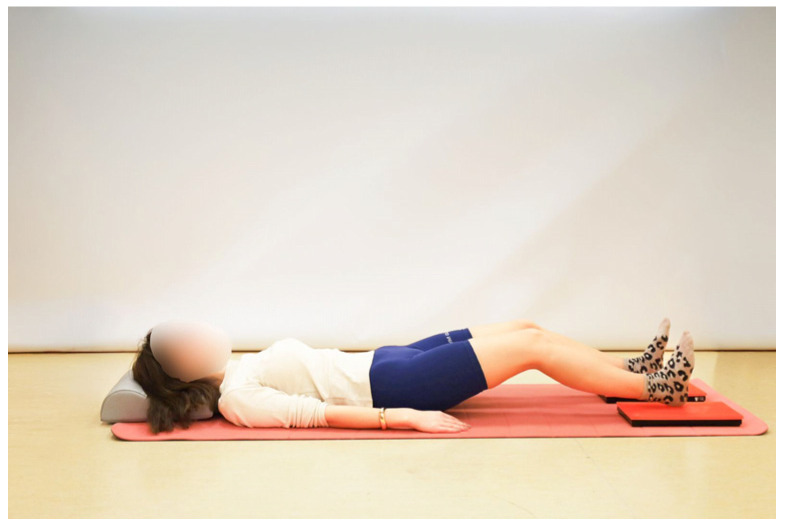
Force plate isometric test (FP-IM-30-Floor) setup: supine position; knee flexed to 30°; heel placed on the force plate positioned on the floor.

For the purposes of the force plate-based tests FP-IM-90/90, FP-IM-30-30 cm, and FP-IM-30-Floor, two force plates, one for each limb, were used (K-Force Plates, Kinvent, Montpellier, France). During measurements, force plates were placed under the heel of the tested limb while participants performed a 3 s isometric knee flexor contraction with maximum effort. One unrecorded familiarization trial preceded data collection. Two maximal trials per condition were recorded. Rest intervals between repetitions and conditions were 120 s. The three testing conditions are presented in Figure 1, Figure 2 and Figure 3.

During the dynamometer-based testing, the dynamometer axis was aligned with the lateral femoral epicondyle, and the lever arm was uniformly set at 40 cm for all participants. The pelvis and the tested limb were stabilized with fixation straps.

The static dynamometer-based test (SD-IM-30) was carried out using a fixed-frame static dynamometer (UPR-1, SUMER, Opole, Poland). Two maximal isometric contractions, each lasting 6 s and preceded by one unrecorded familiarization trial, were conducted, with a 120 s rest interval between trials.

The isokinetic dynamometer-based tests, specifically ID-IK-300, ID-IK-180, and ID-IK-60, were performed using an isokinetic dynamometer (Biodex System 4 Pro, Biodex Medical Systems, Inc., Shirley, NY, USA). Gravity correction was applied according to the manufacturer’s recommendations prior to data collection. Before testing at each angular velocity, participants performed one unrecorded familiarization repetition, followed by a 10 s interval before the recorded set. Recorded sets consisted of 15 repetitions at 300°/s, 10 repetitions at 180°/s, and 5 repetitions at 60°/s, with 120 s rest intervals between successive sets. For each testing condition and limb, the highest recorded value was used for analysis: maximal force for force plate assessments, maximal isometric torque for static dynamometry, and peak torque for isokinetic dynamometry.

For each testing condition and limb, the highest recorded value was used for analysis: maximal force for force plate assessments, maximal isometric torque for static dynamometry, and peak torque for isokinetic dynamometry. These values were subsequently normalized to body mass and expressed in kgf/kg for force plate assessments and Nm/kg for dynamometry-based tests.

The testing procedures used in the present study were based on previously described methods for assessing knee flexor strength. The previous literature indicates that force plate-based isometric hamstring assessments and isokinetic dynamometry can provide reliable measurements of knee flexor strength when standardized testing procedures are applied [41,42,43].

### 2.5. Bias

To limit selection bias, predefined inclusion and exclusion criteria were applied, and all participants underwent a standardized screening procedure prior to enrolment. All participants were recruited using identical eligibility criteria. Only individuals who met all criteria and successfully completed all testing conditions were included in the final analysis.

Measurement bias was reduced through standardized testing protocols, calibrated equipment, and consistent procedures for all participants. All measurements were conducted by trained examiners following a standardized protocol and under the supervision of an experienced researcher to ensure procedural consistency. In addition, participants were familiarized with all testing procedures and completed practice trials prior to data collection to minimize potential learning effects. As already highlighted, standardized verbal commands (“start” and “stop”) and consistent verbal encouragement were used throughout all testing procedures.

To minimize order- and fatigue-related bias, both the sequence of testing modalities and the order of limb assessment were randomized, and standardized rest intervals were applied between trials and testing conditions.

A relatively homogeneous sample of healthy recreational athletes, including both male and female participants, was selected to reduce potential confounding from injury history, training status, and other health-related factors. Sex was treated as the primary independent variable of interest in the analysis.

Because all participants completed all testing conditions, the risk of attrition bias was negligible.

### 2.6. Study Size

The minimum required sample size was calculated using G*Power software (version 3.1.9.6). A significance level of α = 0.05, statistical power (1 − β) = 0.80, and a medium effect size consistent with the study design (d = 0.5) were assumed. In repeated-measures ANOVA, this corresponds approximately to an effect size of f = 0.25. Additionally, a moderate correlation among repeated measures (ρ = 0.50) and a conservative nonsphericity correction (ε = 0.75) were assumed.

The analysis indicated that at least 22 participants were required in each group (separately for females and males). To minimize the risk of incomplete datasets, recruitment of more participants than the power analysis indicated was planned. Accordingly, the target sample size for each group was increased to 26, resulting in a total planned sample of 52.

### 2.7. Quantitative Variables

All quantitative variables, including body mass-normalized knee flexor strength values and LSI, were treated as continuous variables. Strength outcomes were derived from the highest recorded value obtained for each testing condition and limb and corresponded to maximal force for force plate assessments, maximal isometric torque for static dynamometry, and peak torque for isokinetic dynamometry. Body mass-normalized strength values were expressed in kgf/kg for force plate-based tests and in Nm/kg for static and isokinetic dynamometer-based assessments. For each testing condition, LSI was calculated as described earlier in the manuscript.

No categorization or grouping of continuous variables was performed. All analyses were conducted using the original continuous values. Testing condition was treated as a within-subject factor in comparative analyses of normalized strength and LSI outcomes.

### 2.8. Protocol Amendment

One modification to the original recruitment protocol was introduced during the study. Initially, the inclusion criteria specified a BMI range of 18.5–24.99 kg/m^2^. However, due to difficulties recruiting sufficient eligible participants within this range, the upper BMI threshold was extended to 29.99 kg/m^2^. This adjustment was implemented to facilitate recruitment while still limiting the sample to individuals without obesity.

### 2.9. Statistical Methods

Statistical analyses were performed using IBM SPSS Statistics version 30.0.0.0 (172). Descriptive statistics are presented as mean ± standard deviation. Normality of distribution was assessed using the Shapiro–Wilk test, and homogeneity of variance was evaluated using Levene’s test.

Because strength outcomes obtained under different testing conditions were expressed in different physical units (kgf/kg and Nm/kg), standardized effect sizes (Hedges’ g) were also calculated separately for each testing condition to facilitate interpretation of the magnitude of sex-related differences, independent of the original measurement units. Hedges’ g was selected to reduce small-sample bias. Positive values indicate higher normalized strength values in males than in females. Effect sizes were interpreted according to Cohen’s criteria as small (0.2), moderate (0.5), and large (0.8).

To examine sex-related differences in normalized knee flexor strength and LSI values across seven testing conditions, and to assess whether the magnitude of sex-related differences varied across testing conditions, a mixed-design analysis of variance (ANOVA) was performed, with testing condition (seven levels) as a within-subjects factor and sex (female and male) as a between-subjects factor. Separate analyses were conducted for dominant- and non-dominant-limb strength values and for LSI values. When the assumption of sphericity was violated, the Greenhouse–Geisser correction was applied.

Post hoc comparisons were performed using Bonferroni adjustment to control for Type I error inflation. Effect sizes are reported as partial eta squared (ηp^2^) and were interpreted using conventional benchmarks proposed by Cohen, where values of 0.01, 0.06, and 0.14 indicate small, medium, and large effects, respectively.

To address the secondary aim, the strength and direction of linear relationships between normalized strength outcomes across testing conditions, separately for female and male participants and for dominant and nondominant limbs, were calculated using Pearson correlation coefficients (r). The magnitudes of all of the bivariate associations were classified as negligible (0.00–0.30), low (0.31–0.50), moderate (0.51–0.70), high (0.71–0.90), and very high (0.91–1.00).

Because all strength variables were normalized to body mass to account for inter-individual differences in body size, no additional covariate adjustment was applied.

Statistical significance was set at *p* < 0.05.

## 3. Results

A total of 52 participants were included in the study, comprising 26 females and 26 males. All participants met the eligibility criteria and completed all planned testing procedures. Therefore, no participants were excluded after enrollment, and no missing data were recorded for the variables of interest. As all measurements were performed during a single testing session, no follow-up period was applicable.

The characteristics of the study participants are presented in Table 2. Female and male groups were similar in age, whereas males had greater body mass and height than females.

### 3.1. Sex-Related Differences in Normalized Strength Outcomes and LSI Across Testing Conditions

Descriptive statistics for normalized knee flexor strength values in the dominant limb are presented in Table 3.

Descriptive statistics for normalized knee flexor strength values in the non-dominant limb are presented in Table 4. As with the dominant limb, males demonstrated higher mean values than females across all testing conditions.

To facilitate interpretation of sex-related differences across testing conditions expressed in different physical units, standardized effect sizes (Hedges’ g) were calculated separately for each testing condition (Table 5). Effect sizes ranged from small to moderate for the force plate-based assessments (g = 0.20–0.45), from moderate to large for ID-IK-300 (g = 0.49–0.78) and were consistently large for SD-IM-30, ID-IK-180, and ID-IK-60 (g = 1.04–1.42). These findings indicate that the magnitude of sex-related differences was not uniform across testing conditions and tended to be greater in static and slower-velocity dynamometer-based assessments than in force plate-based tests.

A mixed-design ANOVA revealed a significant main effect of testing condition for the dominant limb (Greenhouse–Geisser corrected: F(2.52, 126.14) = 444.09, *p* < 0.001, ηp^2^ = 0.899), indicating that normalized strength outcomes differed across testing conditions. A significant main effect of sex was also observed (F(1, 50) = 25.10, *p* < 0.001, ηp^2^ = 0.334), with males demonstrating higher normalized strength values than females. The interaction effect was also significant (Greenhouse–Geisser-corrected: F(2.52, 126.14) = 14.97, *p* < 0.001, ηp^2^ = 0.230), indicating that the magnitude of sex-related differences varied across testing conditions. Post hoc pairwise comparisons with Bonferroni adjustment showed statistically significant differences between most testing conditions (Bonferroni-adjusted *p* < 0.05). However, because force plate-based and dynamometer-based assessments yield outcomes expressed in different physical units (kgf/kg and Nm/kg), these comparisons should be interpreted with caution and not treated as direct comparisons of absolute strength magnitude across testing modalities. Rather, these findings should be viewed as descriptive information regarding differences in outcomes obtained under the various testing conditions. No significant differences were observed between FP-IM-30-30 cm and FP-IM-30-Floor (*p* = 0.063), nor between SD-IM-30 and ID-IK-60 (*p* = 1.000) after Bonferroni adjustment. All other pairwise comparisons were statistically significant (Bonferroni-adjusted *p* < 0.05). Examination of estimated marginal means confirmed higher normalized strength values in males than in females across all testing conditions.

A mixed-design ANOVA revealed a significant main effect of testing condition for the non-dominant limb (Greenhouse–Geisser corrected: F(2.81, 140.52) = 413.71, *p* < 0.001, ηp^2^ = 0.892), indicating that normalized strength outcomes differed across testing conditions. A significant main effect of sex was also observed (F(1, 50) = 29.45, *p* < 0.001, ηp^2^ = 0.371), with males demonstrating higher normalized strength values than females. Similarly, a significant interaction effect was observed (Greenhouse–Geisser-corrected: F(2.81, 140.52) = 13.73, *p* < 0.001, ηp^2^ = 0.215), indicating that the magnitude of sex-related differences varied across testing conditions. Bonferroni-adjusted post hoc comparisons indicated statistically significant differences between most testing conditions (Bonferroni-adjusted *p* < 0.05). However, because force plate-based and dynamometer-based assessments produce outcomes expressed in different physical units (kgf/kg and Nm/kg), these comparisons should be interpreted with caution and should not be considered direct comparisons of absolute strength magnitude across testing modalities. Rather, these findings should be viewed as descriptive information regarding differences in outcomes obtained under the various testing conditions. No significant differences were observed between FP-IM-90/90 and FP-IM-30-30 cm (*p* = 1.000), nor between SD-IM-30 and ID-IK-180 (*p* = 0.639) after Bonferroni adjustment. All other pairwise comparisons were statistically significant (Bonferroni-adjusted *p* < 0.05). Consistent with the findings for the dominant limb, examination of estimated marginal means confirmed higher normalized strength values in males than in females across all testing conditions.

Consequently, Table 6 presents descriptive statistics for LSI values across testing modalities in females and males. Overall, LSI values were comparable between females and males across all testing conditions.

A mixed-design ANOVA revealed no significant main effect of testing modality for LSI values (Greenhouse–Geisser corrected: F(4.35, 217.43) = 1.05, *p* = 0.385, ηp^2^ = 0.021), indicating comparable symmetry across all testing conditions. No significant main effect of sex was observed (F(1, 50) = 0.02, *p* = 0.896, ηp^2^ < 0.001), suggesting similar LSI values between females and males. Furthermore, no significant interaction between sex and testing condition was found (Greenhouse–Geisser corrected: F(4.35, 217.43) = 0.58, *p* = 0.693, ηp^2^ = 0.011). Post hoc pairwise comparisons with Bonferroni adjustment revealed no significant differences among the testing conditions (all *p* ≥ 0.924). Examination of estimated marginal means confirmed that LSI values were consistent across testing conditions and comparable between sexes.

### 3.2. Correlations Between Normalized Strength Outcomes Obtained Under Different Testing Conditions

**Figure 4:**
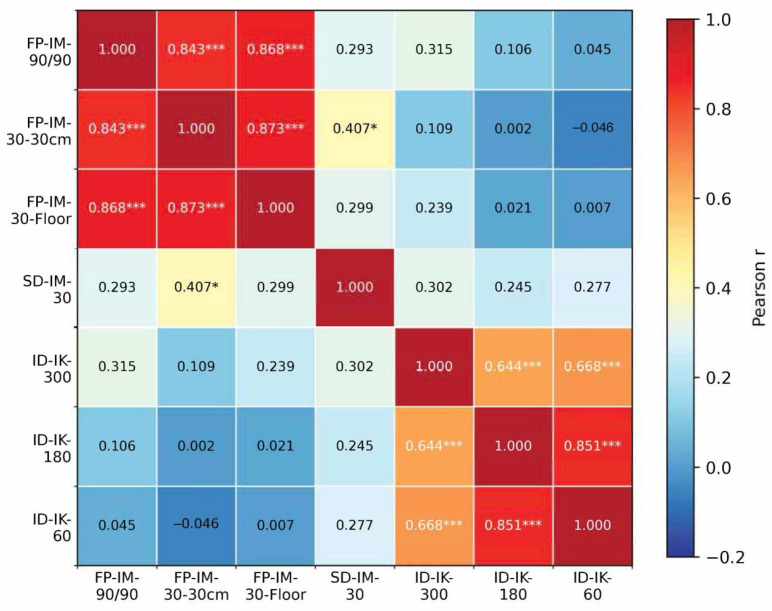
Correlation matrix for normalized knee flexor strength outcomes across testing conditions in the dominant limb in female participants. * *p* < 0.05, *** *p* < 0.001.

**Figure 5:**
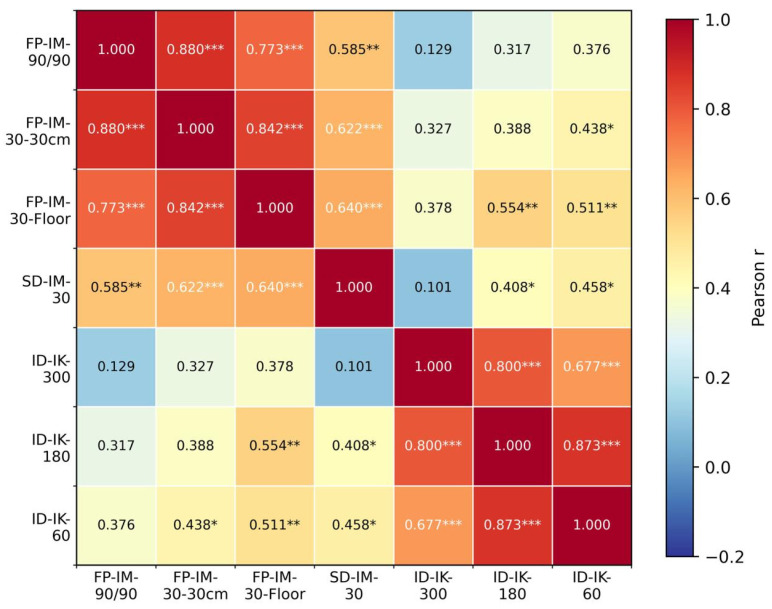
Correlation matrix for normalized knee flexor strength outcomes across testing conditions in the dominant limb in male participants. * *p* < 0.05, ** *p* < 0.01, *** *p* < 0.001.

Figure 4 and Figure 5 present Pearson correlation matrices for normalized knee flexor strength outcomes obtained under the seven testing conditions in the dominant limb of female and male participants, respectively.

In females (Figure 4), high correlations were observed among the three force-plate-based assessments (r = 0.843–0.873, *p* < 0.001). Correlations between the static dynamometer-based assessment and the force plate-based assessments were negligible to low (r = 0.293–0.407), and correlations between the static and isokinetic dynamometer-based assessments were negligible to low (r = 0.245–0.302). Correlations among the three isokinetic dynamometer-based assessments ranged from moderate to high (r = 0.644–0.851, *p* < 0.001). Correlations between the force plate-based and isokinetic dynamometer-based assessments were negligible to low (r = −0.046 to 0.315), indicating limited consistency between these testing groups.

In males (Figure 5), high correlations were observed among the three force-plate-based assessments (r = 0.773–0.880, *p* < 0.001). Correlations between the static dynamometer-based assessment and the force-plate-based assessments were moderate (r = 0.585–0.640, *p* ≤ 0.002). Correlations between the static and isokinetic dynamometer-based assessments were negligible to low (r = 0.101–0.458), whereas correlations among the three isokinetic dynamometer-based assessments ranged from moderate to high (r = 0.677–0.873, *p* < 0.001). Correlations between the force-plate-based and isokinetic dynamometer-based assessments ranged from negligible to moderate (r = 0.129–0.554), suggesting greater consistency across testing groups than in females.

**Figure 6:**
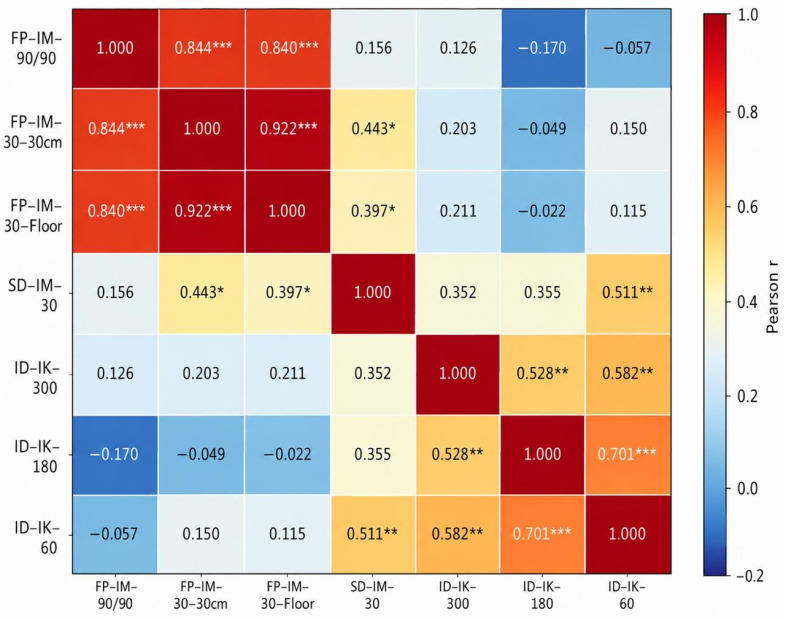
Correlation matrix for normalized knee flexor strength outcomes across testing conditions in the non-dominant limb in female participants. * *p* < 0.05, ** *p* < 0.01, *** *p* < 0.001.

**Figure 7:**
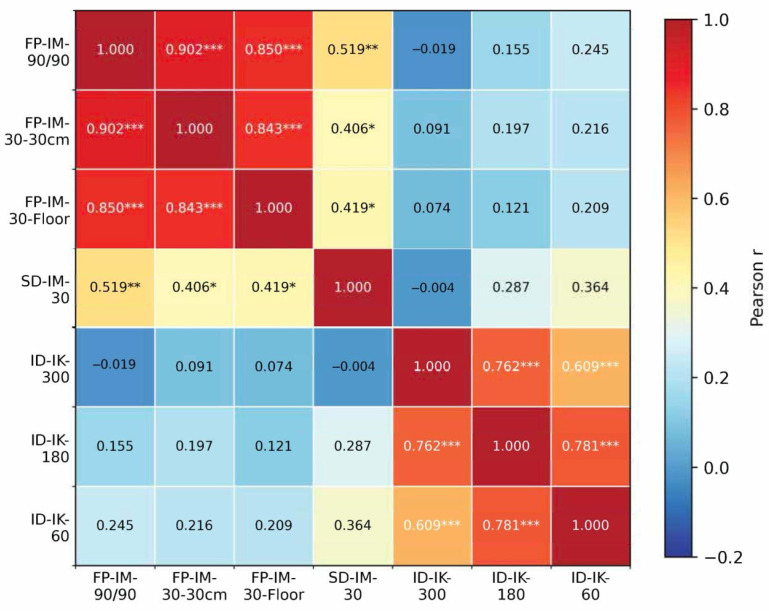
Correlation matrix for normalized knee flexor strength outcomes across testing conditions in the non-dominant limb in male participants. * *p* < 0.05, ** *p* < 0.01, *** *p* < 0.001.

As presented in Figure 6 and Figure 7, Pearson correlation analyses for the nondominant limb showed a similar pattern. In females (Figure 6), correlations among the three force-plate-based assessments were high to very high (r = 0.840–0.922, *p* < 0.001). Correlations between the static dynamometer-based assessment and the force-plate-based assessments were negligible to low (r = 0.156–0.443), and correlations between the static and isokinetic dynamometer-based assessments ranged from low to moderate (r = 0.352–0.511). Correlations among the three isokinetic dynamometer-based assessments were moderate (r = 0.528–0.701, *p* ≤ 0.006). Correlations between the force-plate-based and isokinetic dynamometer-based assessments were generally negligible (r = −0.170 to 0.211).

In males (Figure 7), correlations among the three force plate-based assessments were high to very high (r = 0.843–0.902, *p* < 0.001). Correlations between the static dynamometer-based assessment and the force plate-based assessments ranged from low to moderate (r = 0.406–0.519, *p* < 0.05), and correlations between the static and isokinetic dynamometer-based assessments ranged from negligible to low (r = −0.004 to 0.364). Correlations among the three isokinetic dynamometer-based assessments ranged from moderate to high (r = 0.609–0.781, *p* < 0.001). Correlations between the force plate-based and isokinetic dynamometer-based assessments were generally negligible (r = −0.019 to 0.245).

Overall, high associations were observed within the same group of testing conditions. In contrast, correlations involving the static dynamometer-based assessment and those between different groups of testing conditions were generally weaker and more variable, particularly in female participants. The weakest association was observed between SD-IM-30 and FP-IM-90/90 in females, with a negligible correlation. This finding should be interpreted with caution, as the two tests differ substantially in body position, hip and knee joint configurations, and force-application mechanics. Consequently, they may capture partially different aspects of knee flexor performance and may involve different muscle recruitment strategies and relative contributions of individual hamstring muscles [44]. In addition, the relatively small sample size may have contributed to variability in the correlation estimates. Therefore, the observed weak association should not be interpreted as evidence of reduced validity of either assessment method but rather as reflecting methodological differences between the testing conditions.

## 4. Discussion

The findings of the present study were largely consistent with the proposed hypotheses. Normalized knee flexor strength differed significantly between females and males across all testing conditions, with males demonstrating higher values than females. Standardized effect sizes further indicated that the magnitude of sex-related differences was not uniform across testing conditions, with larger effects observed in static and slower-velocity dynamometer-based assessments than in force plate-based assessments. In contrast, LSI values did not differ significantly between sexes and remained consistent across testing conditions, suggesting that inter-limb symmetry is a relatively stable characteristic in healthy recreational athletes. Regarding the secondary aim, associations between strength outcomes obtained under different testing conditions were generally strongest within the same assessment group, whereas relationships between mechanically different testing approaches were more variable.

### 4.1. Sex-Related Differences Across Testing Conditions

An important methodological consideration is that force plate assessments and dynamometer-based assessments provide outcomes expressed in different physical units (kgf/kg and Nm/kg, respectively). Therefore, direct quantitative comparisons of absolute values between these testing approaches should be interpreted with caution. In the present study, the primary interest was not the comparison of force and torque magnitudes per se but rather the evaluation of sex-related differences across commonly used testing conditions. Examination of standardized effect sizes indicated that sex-related differences were not uniform across testing conditions, with relatively small effects observed in force plate assessments and substantially larger effects observed in static and slower-velocity dynamometer-based assessments.

The results are consistent with the previous literature and reflect expected biological differences between sexes. Higher normalized knee flexor strength was observed in males than in females across all tests, consistent with studies reporting sex-related differences in the structure and function of knee flexor muscles [45].

Previous studies have reported sex-related differences in both the size of the knee flexor muscles and the ratio of knee flexor to knee extensor torque. Greater muscle mass in men has been associated with higher knee flexor torque. In women, certain knee flexor muscles, particularly the sartorius and gracilis, are proportionally smaller relative to the knee extensors, which may contribute to a lower knee flexor-to-extensor torque ratio [45].

From a clinical perspective, lower normalized knee flexor strength in women may be associated with neuromuscular characteristics linked to reduced dynamic knee stability and an increased risk of ACL injury. These differences should be considered in clinical practice when assessing functional muscle strength, developing injury prevention strategies, and individualizing training and rehabilitation programs. Establishing sex-specific reference values may enable more accurate interpretation of risk in women, rather than relying solely on data derived from men [29,31,32,45,46].

### 4.2. Associations Among Strength Outcomes Obtained Under Different Testing Conditions

Among both females and males, the strongest correlations were observed within the same group of testing conditions, whereas associations between different assessment groups were generally weaker and more variable. Descriptive examination of the correlation matrices suggested some differences in association patterns between females and males; however, formal statistical comparisons between sex-specific correlation coefficients were beyond the scope of the present study. Therefore, potential sex-related differences in correlation patterns should be interpreted cautiously.

Importantly, these observations are consistent with a related study conducted in healthy recreationally active males, which demonstrated comparable LSI values across different knee flexor testing conditions, despite only moderate and variable correlations between strength outcomes obtained using different assessment approaches [38]. Together, these findings suggest that different testing conditions may assess related, but not directly interchangeable, aspects of knee flexor performance.

According to the results of the present study, strength outcomes obtained under different testing conditions were generally correlated in both sexes, although the magnitude of these associations varied across testing conditions. This highlights the importance of considering the testing condition when interpreting knee flexor strength data [18,21,26,47,48].

### 4.3. Clinical Relevance of LSI Stability Across Testing Conditions

The relative stability of LSI observed in this study supports previous findings indicating that this metric is largely unaffected by sex despite differences in absolute strength [6]. Clinically, LSI is commonly used as one of the criteria supporting RTS decision-making following ACL reconstruction, with a threshold of ≥90% frequently cited in the literature. However, the present study was conducted in healthy recreational athletes and used the dominant limb as the reference limb. Therefore, the findings should be interpreted as describing physiological inter-limb symmetry rather than supporting the application of specific clinical thresholds. Moreover, current rehabilitation frameworks recommend that LSI should be interpreted alongside strength, functional, and patient-reported outcomes rather than as a standalone criterion [9,10,13,49,50,51].

The present study included healthy recreational athletes, and, in this population, the relative stability of the LSI may reflect physiological limb symmetry, providing a reference for interpreting clinical data. These findings are consistent with those of Leister et al. (2018), who established reference values for the LSI in healthy adults under both fatigued and non-fatigued conditions [52]. The mean LSI was 98.8%, and the analysis revealed no significant sex differences, further supporting the stability of limb symmetry in healthy individuals [52].

Taken together, these findings support the interpretation of LSI as a relatively stable measure of inter-limb symmetry in healthy recreational athletes. The absence of sex-related differences and the consistency of LSI values across testing conditions suggest that physiological limb symmetry is largely preserved regardless of the strength assessment approach used. However, LSI should be interpreted alongside absolute strength outcomes, as symmetry indices alone may not fully reflect underlying strength capacity.

### 4.4. Limitations

Several limitations of the present study should be acknowledged. The analysis was conducted in a cohort of healthy, recreationally active young adults; therefore, the findings cannot be directly generalized to clinical populations or individuals with lower-limb injuries. Nevertheless, understanding how strength and symmetry measures behave in healthy individuals provides a valuable reference for future clinical and rehabilitation research. Also, the relative homogeneity of the sample with respect to age, body composition, and activity level may limit the applicability of the results to other populations, such as elite athletes or older adults. Furthermore, the cross-sectional design precludes conclusions regarding changes in strength and limb symmetry over time or in response to training and rehabilitation.

An additional limitation relates to participant recruitment and sample definition. Although most participants were recruited from the medical university, additional recruitment outside the university was necessary to reach the target sample size. Furthermore, the upper BMI inclusion threshold was expanded during recruitment from 24.99 to 29.99 kg/m^2^ to facilitate enrollment.

Although all strength outcomes were normalized to body mass, this approach does not fully account for potential sex-related differences in body composition, lean mass distribution, or limb segment dimensions. Consequently, some of the observed differences between females and males may reflect anthropometric and morphological factors that were not directly assessed in the present study.

A further limitation relates to the methodological characteristics of the testing procedures. Because force plate- and dynamometer-based assessments yield outcomes expressed in different physical units, direct comparisons of absolute outcome magnitude between testing conditions are inherently limited. In addition, although the order of testing modalities and the order of assessed limbs were randomized for each participant to minimize potential learning and fatigue effects, the present study was not designed to evaluate sequence-related differences. Therefore, a formal statistical analysis of sequence effects could not be performed retrospectively, and residual order- or fatigue-related influences cannot be completely excluded.

Furthermore, the correlation analyses were exploratory. Although associations were examined separately in female and male participants, formal statistical comparisons between sex-specific correlation coefficients were not performed. Therefore, potential sex-related differences in correlation patterns should be interpreted cautiously.

In addition, the limb symmetry index is a calculated metric that may mask bilateral strength deficits and should therefore be interpreted alongside absolute strength values.

### 4.5. Implications of the Findings

The findings of the present study extend current knowledge by providing sex-specific data on normalized knee flexor strength and limb symmetry across multiple testing conditions in healthy recreational athletes. The results emphasize the importance of considering sex as a biological variable and indicate that strength outcomes obtained using different testing conditions should not be interpreted as directly interchangeable. Findings of the present study may serve as reference values for future research and support the interpretation of knee flexor strength assessments in similar populations.

## 5. Conclusions

The present study demonstrated that normalized knee flexor strength differed between females and males across all testing conditions. Standardized effect sizes suggested that the magnitude of sex-related differences was not uniform across testing conditions, with larger sex-related effects identified in static and slower-velocity dynamometer-based assessments than in force plate-based assessments. In contrast, LSI values remained consistent across testing conditions and did not differ between sexes. Associations between strength outcomes obtained under different testing conditions were generally strongest within the same assessment group. Descriptive analyses suggested that the pattern of associations among testing conditions may differ between females and males; however, these observations should be interpreted as exploratory, as formal statistical comparisons of sex-specific correlation coefficients were not performed. These findings indicate that both sex and testing methodology should be considered when interpreting knee flexor strength outcomes, whereas limb symmetry appears to be a relatively stable characteristic in healthy recreational athletes.

## Figures and Tables

**Table 1:** Testing setup and positioning for knee flexor strength assessments.

Testing Condition	Contraction Type	Device	Body Position	Tested Knee Joint Position	Device Position
FP-IM-90/90	Isometric	Force plate	Supine	90°	On the elevated support
FP-IM-30-30 cm	Isometric	Force plate	Supine	30°	On the 30 cm support
FP-IM-30-Floor	Isometric	Force plate	Supine	30°	On the floor
SD-IM-30	Isometric	Static dynamometer	Prone	30°	Standard setup
ID-IK-300	Isokinetic	Isokinetic dynamometer	Seated	Full ROM	Standard setup
ID-IK-180	Isokinetic	Isokinetic dynamometer	Seated	Full ROM	Standard setup
ID-IK-60	Isokinetic	Isokinetic dynamometer	Seated	Full ROM	Standard setup

Abbreviations: FP-IM-90/90, force plate isometric test with hip and knee flexed to 90°; FP-IM-30-30 cm, force plate isometric test at 30° knee flexion with the force plate positioned on a 30 cm support; FP-IM-30-Floor, force plate isometric test at 30° knee flexion with the force plate positioned on the floor; SD-IM-30, static dynamometer isometric test at 30° knee flexion; ID-IK-300/180/60, isokinetic dynamometer tests performed at 300°/s, 180°/s, and 60°/s, respectively; ROM, range of motion.

**Table 2:** Characteristics of the females and males studied.

Variable	Female (*n* = 26)	Male (*n* = 26)
Age (years)	23.85 ± 2.17	23.19 ± 2.26
Body mass (kg)	61.23 ± 6.88	81.19 ± 8.15
Body height (cm)	166.19 ± 6.52	184.31 ± 6.44
Body mass index (kg/m^2^)	22.16 ± 2.07	23.89 ± 1.92

Values expressed as mean ± standard deviation. Abbreviations: *n*, number of participants.

**Table 3:** Normalized knee flexor strength values in the dominant limb across testing conditions in females and males.

Testing Condition	Female (*n* = 26)	Male (*n* = 26)
FP-IM-90/90 (kgf/kg)	0.25 ± 0.05	0.27 ± 0.05
FP-IM-30-30 cm (kgf/kg)	0.24 ± 0.05	0.26 ± 0.04
FP-IM-30-Floor (kgf/kg)	0.23 ± 0.05	0.25 ± 0.05
SD-IM-30 (Nm/kg)	0.80 ± 0.19	1.24 ± 0.38
ID-IK-300 (Nm/kg)	0.77 ± 0.21	0.86 ± 0.16
ID-IK-180 (Nm/kg)	0.85 ± 0.16	1.07 ± 0.20
ID-IK-60 (Nm/kg)	1.14 ± 0.21	1.39 ± 0.22

Values expressed as mean ± standard deviation. Abbreviations: *n*, number of participants; FP-IM-90/90, force plate isometric test with hip and knee flexed to 90°; FP-IM-30-30 cm, force plate isometric test at 30° knee flexion with the force plate positioned on a 30 cm support; FP-IM-30-Floor, force plate isometric test at 30° knee flexion with the force plate positioned on the floor; SD-IM-30, static dynamometer isometric test at 30° knee flexion; ID-IK-300/180/60, isokinetic dynamometer tests performed at 300°/s, 180°/s, and 60°/s, respectively.

**Table 4:** Normalized knee flexor strength values in the non-dominant limb across testing conditions in females and males.

Testing Condition	Female (*n* = 26)	Male (*n* = 26)
FP-IM-90/90 (kgf/kg)	0.24 ± 0.05	0.26 ± 0.05
FP-IM-30-30 cm (kgf/kg)	0.24 ± 0.05	0.25 ± 0.05
FP-IM-30-Floor (kgf/kg)	0.23 ± 0.05	0.24 ± 0.05
SD-IM-30 (Nm/kg)	0.82 ± 0.23	1.22 ± 0.33
ID-IK-300 (Nm/kg)	0.74 ± 0.14	0.86 ± 0.17
ID-IK-180 (Nm/kg)	0.81 ± 0.23	1.04 ± 0.19
ID-IK-60 (Nm/kg)	1.08 ± 0.18	1.37 ± 0.26

Values expressed as mean ± standard deviation. Abbreviations: *n*, number of participants; FP-IM-90/90, force plate isometric test with hip and knee flexed to 90°; FP-IM-30-30 cm, force plate isometric test at 30° knee flexion with the force plate positioned on a 30 cm support; FP-IM-30-Floor, force plate isometric test at 30° knee flexion with the force plate positioned on the floor; SD-IM-30, static dynamometer isometric test at 30° knee flexion; ID-IK-300/180/60, isokinetic dynamometer tests performed at 300°/s, 180°/s, and 60°/s, respectively.

**Table 5:** Standardized effect sizes (Hedges’ g) for sex-related differences in normalized knee flexor strength across testing conditions.

Testing Condition	Dominant Limb	Non-Dominant Limb
FP-IM-90/90	0.41	0.43
FP-IM-30-30 cm	0.45	0.20
FP-IM-30-Floor	0.45	0.39
SD-IM-30	1.42	1.37
ID-IK-300	0.49	0.78
ID-IK-180	1.17	1.04
ID-IK-60	1.15	1.29

Values are presented as Hedges’ g standardized effect sizes for the comparison between female and male participants. Abbreviations: FP-IM-90/90, force plate isometric test with hip and knee flexed to 90°; FP-IM-30-30 cm, force plate isometric test at 30° knee flexion with the force plate positioned on a 30 cm support; FP-IM-30-Floor, force plate isometric test at 30° knee flexion with the force plate positioned on the floor; SD-IM-30, static dynamometer isometric test at 30° knee flexion; ID-IK-300/180/60, isokinetic dynamometer tests performed at 300°/s, 180°/s, and 60°/s, respectively.

**Table 6:** Limb symmetry index values across testing conditions in females and males.

Testing Condition	Female (*n* = 26)	Male (*n* = 26)
FP-IM-90/90	95.80 ± 9.68	96.12 ± 8.52
FP-IM-30-30 cm	101.16 ± 9.60	96.28 ± 9.92
FP-IM-30-Floor	98.64 ± 8.83	98.44 ± 12.90
SD-IM-30	102.08 ± 14.73	99.19 ± 14.95
ID-IK-300	100.35 ± 19.58	101.37 ± 15.82
ID-IK-180	96.29 ± 23.64	98.06 ± 11.83
ID-IK-60	95.15 ± 11.19	98.42 ± 12.16

Values expressed as mean ± standard deviation. Abbreviations: *n*, number of participants; FP-IM-90/90, force plate isometric test with hip and knee flexed to 90°; FP-IM-30-30 cm, force plate isometric test at 30° knee flexion with the force plate positioned on a 30 cm support; FP-IM-30-Floor, force plate isometric test at 30° knee flexion with the force plate positioned on the floor; SD-IM-30, static dynamometer isometric test at 30° knee flexion; ID-IK-300/180/60, isokinetic dynamometer tests performed at 300°/s, 180°/s, and 60°/s, respectively.

## Data Availability

The original data presented in the study are openly available on the Polish Platform of Medical Research at https://doi.org/10.60956/vt9h-kb32.

## Abbreviations

The following abbreviations are used in this manuscript:

ACL	Anterior cruciate ligament
BMI	Body mass index
FP-IM-30-30 cm	Force plate isometric test at 30° knee flexion with the force plate positioned on a 30 cm support
FP-IM-30-Floor	Force plate isometric test at 30° knee flexion with the force plate positioned on the floor
FP-IM-90/90	Force plate isometric test with hip and knee flexed to 90°
ID-IK-180	Isokinetic dynamometer tests performed at 180°/s
ID-IK-300	Isokinetic dynamometer tests performed at 300°/s
ID-IK-60	Isokinetic dynamometer tests performed at 60°/s
LSI	Limb symmetry index
RTS	Return to sport
PAL	Physical Activity Level
SD-IM-30	Static dynamometer isometric test at 30° knee flexion
